# In-depth immunophenotyping data of IL-6R on the human peripheral regulatory T cell (Treg) compartment

**DOI:** 10.1016/j.dib.2017.04.043

**Published:** 2017-05-02

**Authors:** Ricardo C. Ferreira, Daniel B. Rainbow, Arcadio Rubio García, Marcin L. Pekalski, Linsey Porter, João J. Oliveira, Frank Waldron-Lynch, Linda S. Wicker, John A. Todd

**Affiliations:** a JDRF/Wellcome Trust Diabetes and Inflammation Laboratory, Wellcome Trust Centre for Human Genetics, Nuffield Department of Medicine, NIHR Oxford Biomedical Research Centre, University of Oxford, Oxford, UK; b JDRF/Wellcome Trust Diabetes and Inflammation Laboratory, Department of Medical Genetics, NIHR Cambridge Biomedical Research Centre, Cambridge Institute for Medical Research, University of Cambridge, Cambridge, UK; c Experimental Medicine and Immunotherapeutics, Department of Medicine, NIHR Cambridge Biomedical Research Centre, University of Cambridge, Cambridge, UK; d NIHR Cambridge Clinical Trial Unit, Cambridge NHS University Hospitals Trust, Cambridge Biomedical Research Centre, University of Cambridge, Cambridge, UK

**Keywords:** IL-6 receptor, Regulatory T cells, Immunophenotyping, Human immunology

## Abstract

We provide in this paper a detailed characterization of the human peripheral CD4^+^ CD127^low^CD25^+^ regulatory T cell (Treg) compartment, with a particular emphasis in defining the population expressing higher levels of the IL-6 receptor (IL-6R). We provide a description of the phenotype of this population by assessing both the surface expression by flow cytometry as well as their transcriptional profile and functional features. In addition, we also present functional data describing the responsiveness of these subsets to IL-6 signalling *in vitro* and to IL-2 *in vivo*. The data presented in this paper support the research article “Human IL-6R^hi^TIGIT^−^ CD4^+^CD127^low^CD25^+^ T cells display potent *in vitro* suppressive capacity and a distinct Th17 profile” (Ferreira RC et al., 2017; doi: 10.1016/j.clim.2017.03.002) [1].

**Specifications Table**

**Table:** 

Subject area	*Biology*
More specific subject area	*Human Immunology*
Type of data	*Tables (x3) and Figures (x12)*
How data was acquired	*Flow cytometry (Fortessa; BD biosciences); Gene expression profiling (NanoString Technologies)*
Data format	*Analyzed flow cytometry files and normalized gene expression counts (from Nanostring)*
Experimental factors	*Flow cytometry and gene expression profiling was performed in freshly isolated PBMCs or CD4^+^ T cells. Cytokine production was assessed following in vitro stimulation with PMA + ionomycin. Cell proliferation was assessed by flow cytometry by culturing cells in vitro with anti-CD3/anti-CD28 stimulation.*
Experimental features	*Delineation of the Treg compartment was performed in human peripheral blood cells using polychromatic flow cytometry. The global transcriptional profile of the assessed T cell subsets was assessed in sorted cells isolated ex vivo.*
Data source location	*Samples from human volunteers and T1D patients were collected in Cambridge, UK.*
Data accessibility	*All primary non-clinical data are available in this article. The DILT1D data from individuals prior to normalization as a group are available, however they cannot be anonymized sufficiently to be able to put into the public domain without risk of participant identification. Data are available on request, through the Cambridge University institutional repository (DOI link:*https://doi.org/10.17863/CAM.832*).*
Related research article	*The data presented in this paper support the research article “Human IL-6R*^*hi*^*TIGIT*^*−*^*CD4*^*+*^*CD127*^*low*^*CD25*^*+*^*T cells display potent in vitro suppressive capacity and a distinct Th17 profile” (Ferreira RC et al., 2017)*[1].

**Value of the data**

• Data provide a detailed description of the human peripheral Treg compartment at both the protein and transcriptional level.
• The flow cytometry data provides a valuable resource for other researchers to compare the expression levels of a number of classical Treg surface markers within the defined CD4^+^ T cell populations defined here.
• The detailed information on the flow cytometry antibodies and polychromatic panel combinations will be a valuable tool for researchers in the field to help design their specific immunostaining panels.
• Cell subset heterogeneity is one of the main challenges in the description of human Treg populations, and is clearly highlighted in the data presented in this paper.
• Data provide precise quantitative information regarding the expression levels of a broad range of immune genes at the mRNA level in highly purified human Treg subsets both *ex vivo* and after *in vitro* stimulation.

## 1 Data

**Fig. 1:**
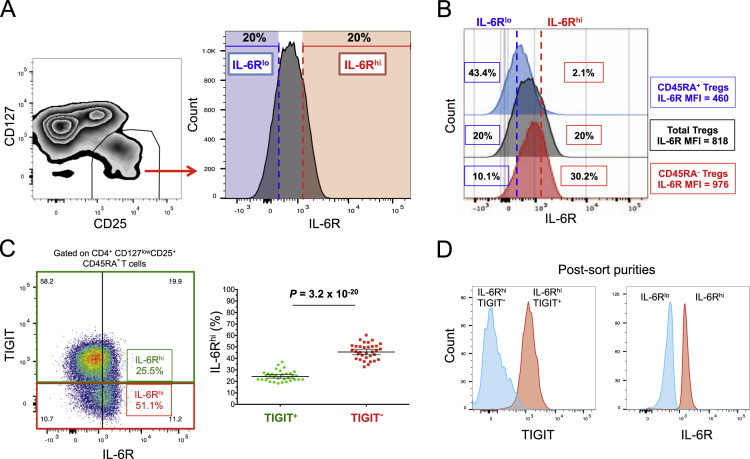
Gating strategy for the delineation of IL-6R^hi^ CD127^low^CD25^+^ T cells (Tregs). (A) Gating strategy for the delineation of IL-6R^lo^ and IL-6R^hi^ CD127^low^CD25^+^ Tregs, defined as the lower and upper 20th percentile, respectively, of the IL-6R mean fluorescence intensity (MFI) distribution in total CD127^low^CD25^+^ Tregs. (B) Histograms depict the distribution of IL-6R expression and the respective frequency of IL-6R^lo^ and IL-6R^hi^ cells among: (i) CD45RA^+^ naïve (depicted in blue); (ii) total (depicted in black); and (iii) CD45RA^−^ memory (depicted in red) CD127^low^CD25^+^ Tregs. Frequencies shown in the figure represent the average frequency of the IL-6R^lo^ and IL-6R^hi^ cells in the three assessed Treg subsets from 33 healthy donors. (C) Data shown depict the expression of TIGIT versus IL-6R in one illustrative donor and the frequency (GeoMean +/− 95% CI) of IL-6R^hi^ cells in the TIGIT^+^ (depicted in green) and TIGIT^−^ (depicted in red) subsets of CD127^low^CD25^+^CD45RA^−^ mTregs in 33 healthy donors. *P* value was calculated using a two-tailed paired non-parametric Wilcoxon signed rank test, comparing the frequency of IL-6R^hi^ cells in the TIGIT^+^ and TIGIT^−^ subsets. (D) Histograms illustrate the distribution of the two sorting markers TIGIT and IL-6R following flow-cytometric sorting.

**Fig. 2:**
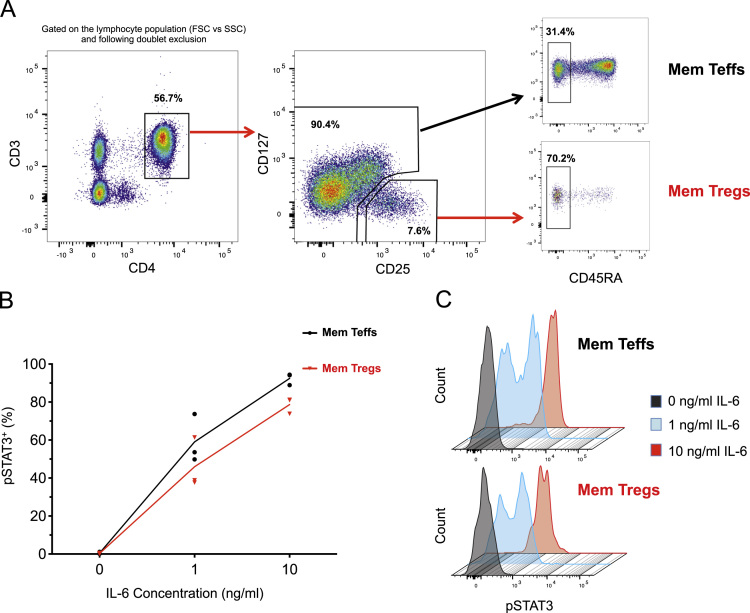
Memory Tregs are sensitive to IL-6 signalling *in vitro*. (A) Gating strategy for the delineation of the CD45RA^−^ memory T effector (Teff) and Treg subsets. (B) Frequency of pSTAT3^+^ cells following stimulation of freshly isolated PBMCs with 0, 1 or 10 ng/ml of IL-6. Intracellular levels of pSTAT3 were measured by flow cytometry in CD4^+^ memory Teffs (depicted in black) and in CD4^+^ memory Tregs (depicted in red) from three healthy volunteers. (C) Histograms depict the distribution of the pSTAT3 mean fluorescence intensity (MFI) in memory Teffs (top panel) and memory Tregs (bottom panel) in response to stimulation with 0, 1 or 10 ng/ml of IL-6 in one illustrative donor.

**Fig. 3:**
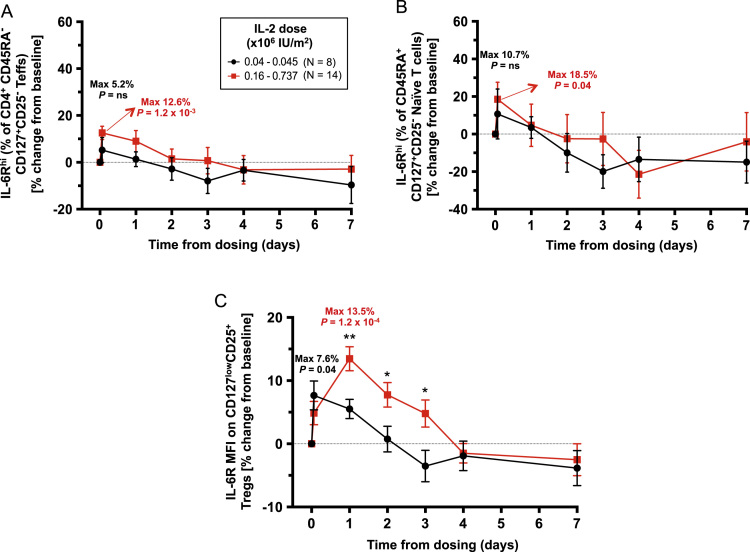
Effect of single-dose IL-2 treatment on the expression of IL-6R on the CD4^+^ T cell compartment *in vivo*. (A,B) Data shown depict the variation (Mean +/− SEM) of the frequency of IL-6R^hi^ cells on: (i) CD127^+^CD25^−^ CD45RA^−^ Teffs (A); and (ii) IL-6R^hi^ CD127^+^CD25^−^ CD45RA^+^ naïve T cells (B) compared to the pre-treatment baseline, following IL-2 treatment in 22 T1D patients enrolled in the “*Adaptive study of IL-2 dose on regulatory T cells in type 1 diabetes”* (DILT1D) [2], [3]. Median pre-treatment baseline frequencies of IL-6R^hi^ cells were 39.3% (range: 21.5–59.9%) and 5.39% (range: 1.49–30.4%) within CD4^+^ CD45RA^−^ Teffs and CD4^+^ CD45RA^+^ naïve T cells, respectively. (C) Data shown depict the variation (Mean +/− SEM) of the IL-6R mean fluorescence intensity (MFI) levels on the surface of total CD127^low^CD25^+^ Tregs compared to the pre-treatment baseline MFI levels (median = 600; range: 392–836). Patients were stratified based on whether they received: (i) the lower IL-2 doses of 0.04–0.045 x 10^6^ U/ml (N = 8; depicted in black); or (ii) the higher IL-2 doses of 0.16–0.737×10^6^ U/ml (N = 14; depicted in red). The MFI cuttoff to define IL-6R^hi^ cells was the same as the one used for the Treg subset (detailed in Fig. 1). The maximum increases over the baseline pre-treatment frequencies achieved during the course of the study are indicated for each IL-2 dosing group. *P* values for the maximum increase in the frequency of the assessed parameter in response to a single dose of IL-2 was calculated using a two-tailed paired non-parametric Wilcoxon signed rank test comparing the frequencies observed at the timepoint where the maximal increase was achieved with the respective baseline pre-treatment frequencies. *P* values for the IL-2 dose-dependent effects were calculated using a two-tailed non-parametric a two-tailed non-parametric Mann-Whitney test comparing the frequency of IL-6R^hi^ cells between the low and high dose groups at each timepoint. The DILT1D data from individuals prior to normalization as a group are available, however they cannot be anonymized sufficiently to be able to put into the public domain without risk of participant identification. Data are available on request, through the Cambridge University institutional repository (DOI link: https://doi.org/10.17863/CAM.832). *P < 0.05; **P < 0.01; ns = not significant.

**Fig. 4:**
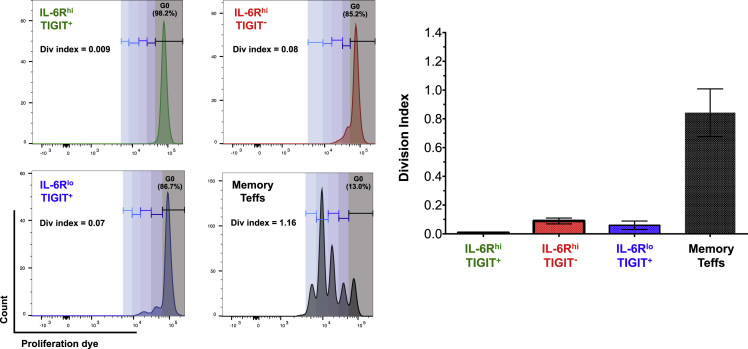
Proliferative capacity of IL-6R^hi^TIGIT^−^ mTregs is dependent on exogenous IL-2. Proliferative capacity of sorted (i) IL-6R^hi^TIGIT^+^ (depicted in green), (ii) IL-6R^hi^TIGIT^−^ (depicted in red), (iii) IL-6R^lo^TIGIT^+^ (depicted in blue) mTregs, and (iv) CD127^+^CD25^−^CD45RA^−^ Teff cells (depicted in black) was assessed in response to *in vitro* stimulation with anti-CD3/CD28 beads, in the absence of exogenous IL-2. Data (mean +/− SEM) were obtained from cells sorted from three independent donors. Proliferation and suppressive capacity were calculated using the Division Index in FlowJo, setting 0% suppression as the condition with the respective Teffs cultured in the absence of Tregs.

**Fig. 5:**
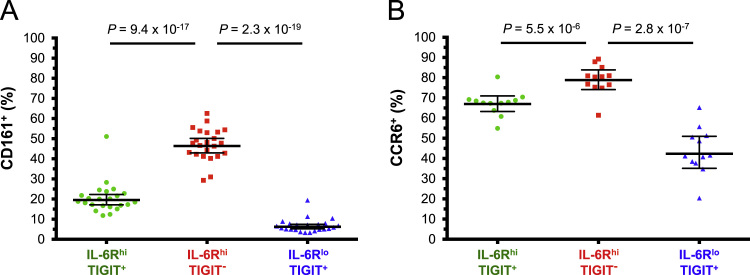
CD161 and CCR6 expression is increased on IL-6R^hi^TIGIT^−^ mTregs. (A,B) Frequency (GeoMean +/− 95% CI) of CD161 (N = 23) (A) and CCR6 (N = 12) (B) was assessed by flow cytometry in freshly isolated PBMCs from healthy donors. Data were stratified according to the three assessed mTreg subsets: IL-6R^lo^ (depicted in blue), IL-6R^hi^TIGIT^+^ (depicted in green) and IL-6R^hi^TIGIT^−^ (depicted in red). *P* values were calculated using a two-tailed paired non-parametric Wilcoxon signed rank test.

**Fig. 6:**
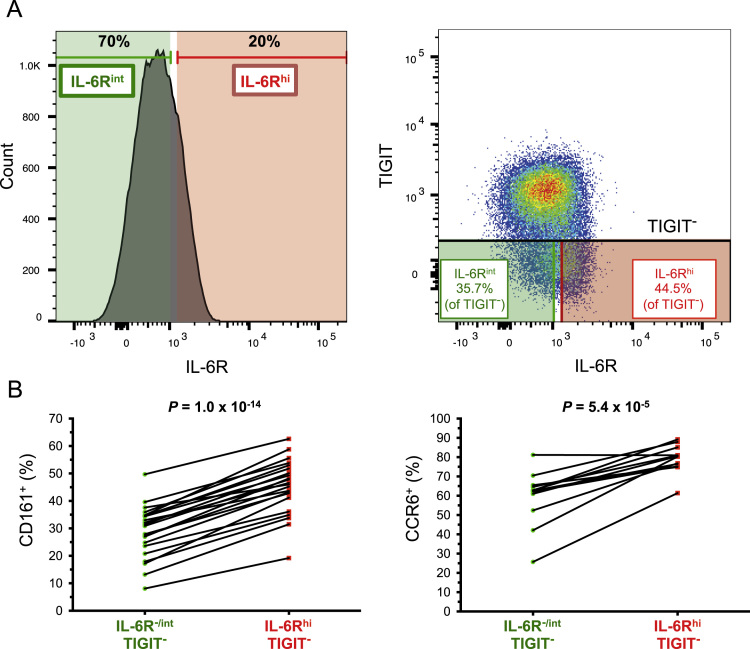
Elevated IL-6R expression delineates a subset of TIGIT^−^ mTregs with increased expression of the canonical Th17 markers CD161 and CCR6. (A) Gating strategy to delineate IL-6R^−/int^ (depicted in green) and IL-6R^hi^ (depicted in red) TIGIT^−^ mTregs. (B) Data shown depict the frequencies of the Th17 markers CD161 (N = 23) and CCR6 (N = 12) in IL-6R^−/int^ and IL-6R^hi^ mTregs, obtained by flow cytometry in freshly isolated PBMCs from healthy donors. *P* values were calculated using a two-tailed paired non-parametric Wilcoxon signed rank test.

**Fig. 7:**
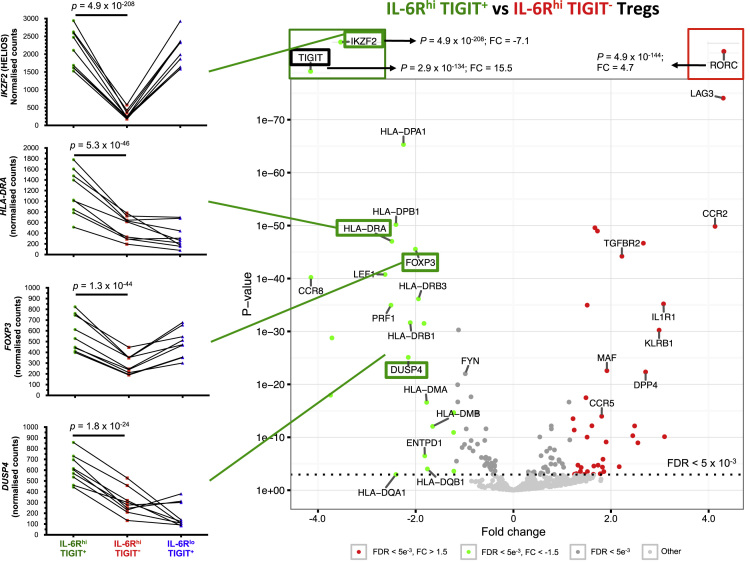
*ex vivo* isolated IL-6R^hi^TIGIT^+^ mTregs show an activated Treg transcriptional profile. Volcano plot depicts the differential expression of 579 immune genes in IL-6R^hi^TIGIT^+^ and IL-6R^hi^TIGIT^−^ mTregs sorted *ex vivo* from nine independent healthy donors using NanoString. Plots depicting the normalized read counts from four differentially expressed activated Treg signature genes (marked in green), *HELIOS*, *HLA-DRA*, *DUSP4* and *FOXP3* are also shown. The flow sorting marker TIGIT, used for the isolation of the assessed Treg subsets, is marked in black. *P* values were calculated using two-tailed paired non-parametric Wilcoxon signed rank tests, comparing the normalized NanoString read counts between IL-6R^hi^TIGIT^+^ and IL-6R^hi^TIGIT^−^ mTregs.

**Fig. 8:**
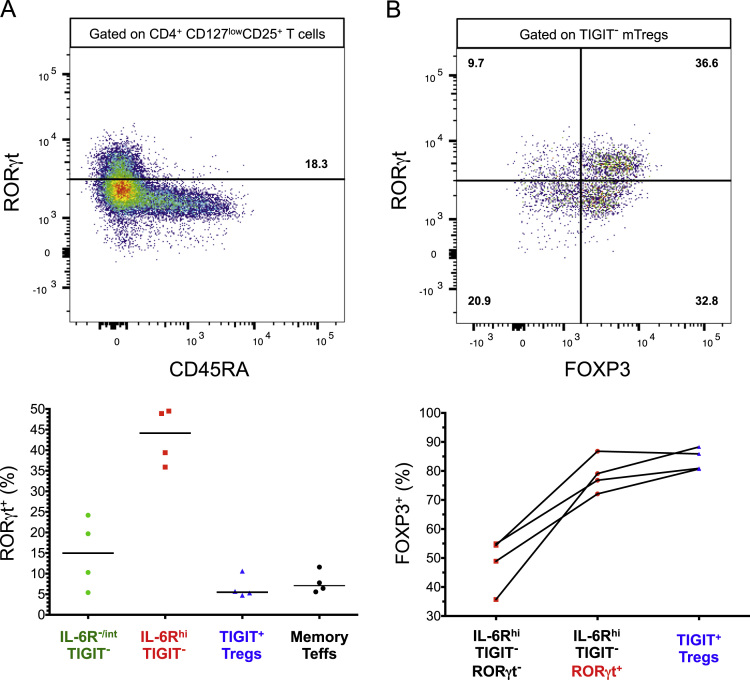
Elevated expression of IL-6R on TIGIT^−^ mTregs marks a subset of FOXP3^+^ RORγt^+^ TIGIT^−^ mTregs. (A) Expression of the canonical Th17 transcription factor RORγt was assessed on four healthy donors using intracellular flow cytometry. Data shown depicts the distribution of RORγt^+^ cells in CD45RA^−^ IL-6R^−/int^TIGIT^−^; IL-6R^hi^TIGIT^−^ and TIGIT^+^ CD127^low^CD25^+^ T cells; and in memory T effector cells (see gating strategy on Fig. 6). The histogram shown depicts an illustrative example of the expression of RORγt (stratified by CD45RA) on total CD4^+^ CD127^low^CD25^+^ T cells. Gating of RORγt^+^ cells as detected by mAb clone AFKJS-9 followed that of Ayyoub M et al. [4]. (B) Frequency of FOXP3^+^ cells was assessed within the RORγt^−^ and RORγt^+^ fractions of IL-6R^hi^TIGIT^−^ mTregs, and in TIGIT^+^ mTregs. Illustrative histogram depicts the co-expression of FOXP3 and RORgt with TIGIT- CD45RA^−^ CD127^low^CD25^+^ T cells. Horizontal bars represent the median distribution of the assessed immune phenotypes in each group.

**Fig. 9:**
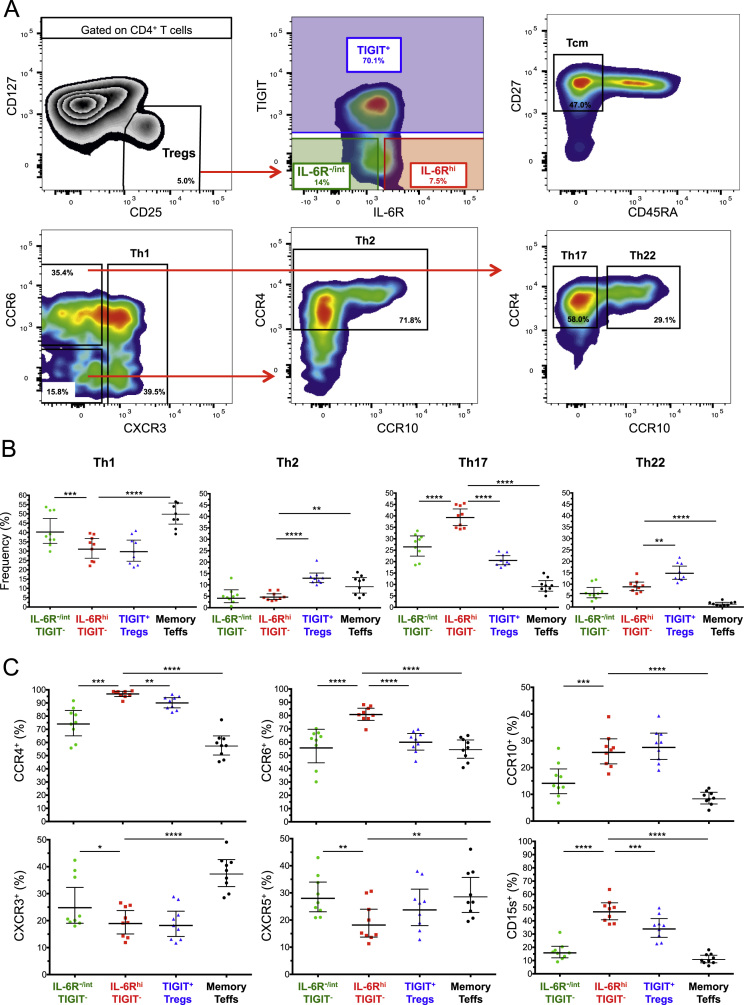
IL-6R^hi^TIGIT^+^ mTregs chemokine receptor profile is consistent with a tissue-homing Treg subset. (A) Gating strategy for the delineation of the Th1, Th2, Th17 and Th22 Treg subsets. The delineation of the Th Treg subsets was based on the definition previously described by Duhen et al. [5]. (B) Data shown depicts the frequencies (GeoMean +/− 95% CI) of the Th subsets in CD45RA^−^ IL-6R^−/int^TIGIT^−^; IL-6R^hi^TIGIT^−^ and TIGIT^+^ CD127^low^CD25^+^ T cells; and in memory T effector cells. (C) Data shown depicts the frequency of the individual chemokine receptors and cell adhesion markers in the same T cell subsets. *P* values were calculated using a two-tailed paired non-parametric Wilcoxon signed rank test, comparing the frequency of the assessed phenotypes between IL-6R^hi^TIGIT^−^ mTregs and the other T cells subsets. *P < 0.05; **P < 0.01; ***P < 0.001; ****P < 10^−^^4^.

**Fig. 10:**
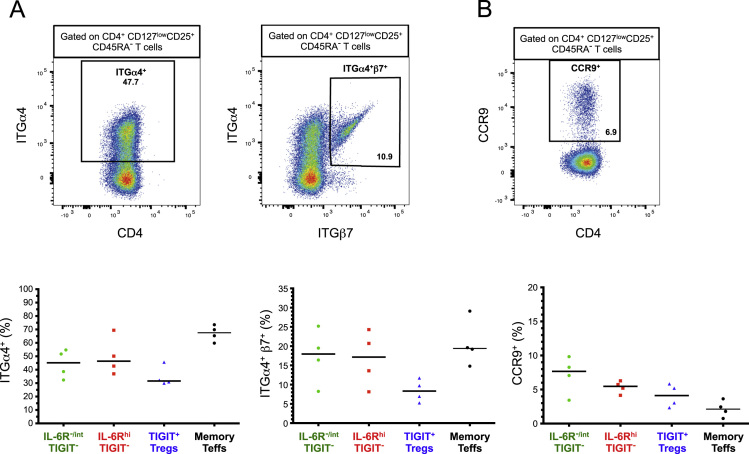
IL-6R^hi^TIGIT^+^ mTregs display the capacity to migrate to the gut. (A,B) Gating strategy for the delineation of the colon-homing receptors ITGα4^+^ and ITGα4^+^β7^+^ cells (A) and the small intestine-homing receptor CCR9^+^ cells (B). Illustrative plots depict the expression of the assessed gut-homing receptors on CD45RA^−^ CD4^+^ CD127^low^CD25^+^ T cells (C) Data shown depicts the distribution (median) of ITGα4^+^, ITGα4^+^β7^+^ and CCR9^+^ cells in CD45RA^−^ IL-6R^−/int^TIGIT^−^; IL-6R^hi^TIGIT^−^ and TIGIT^+^ CD127^low^CD25^+^ T cells; and in memory T effector cells.

**Fig. 11:**
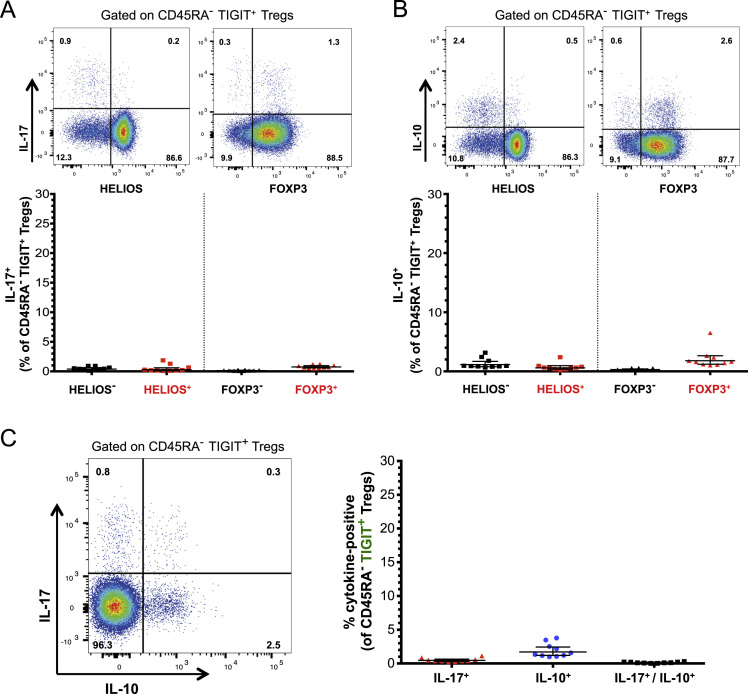
CD45RA^−^ TIGIT^+^ memory Tregs produce limited amounts of IL-17 and IL-10 upon *in vitro* activation. (A,B) Data shown depict the frequency (GeoMean +/- 95% CI) of IL-17^+^ (A) and IL-10^+^ (B) cells among CD45RA^−^TIGIT^+^ mTregs, stratified by the expression of HELIOS and FOXP3. IL-17 and IL-10 production was assessed by intracellular flow cytometry in freshly isolated PBMCs from 10 healthy donors, following *in vitro* activation with PMA + ionomycin. (C) Data depict the frequency (GeoMean +/− 95% CI) of IL-17 and IL-10 single-producers as well as IL-17/IL-10 double producers among the CD45RA^−^TIGIT^+^ Treg subset.

**Fig. 12:**
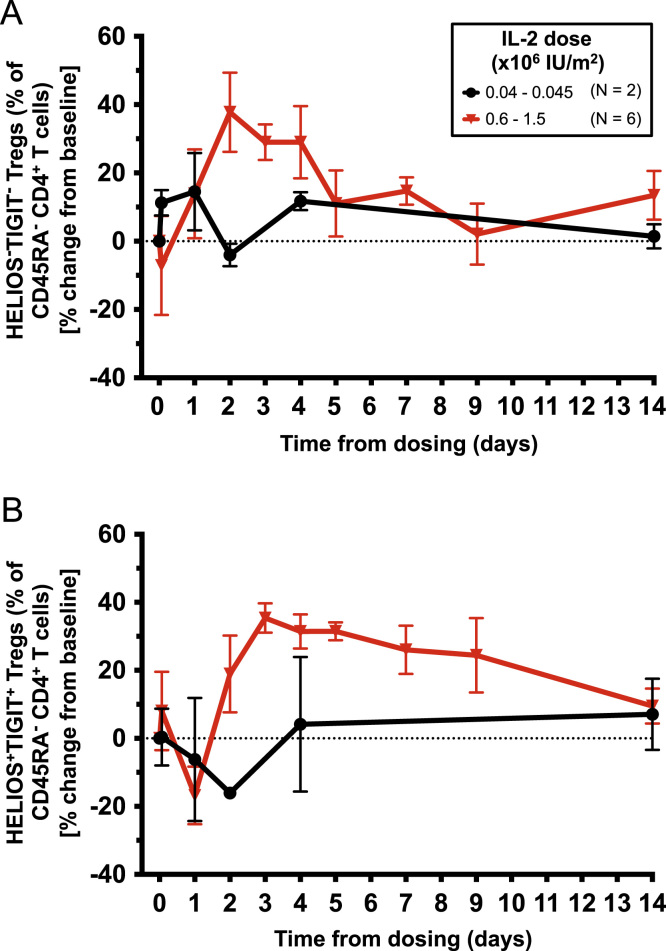
Single low dose of IL-2 does not preferentially expand CD45RA^−^ HELIOS^−^TIGIT^−^ mTregs *in vivo*. (A,B) Data depict the variation (Mean +/− SEM) of the frequency of CD45RA^−^ HELIOS^−^TIGIT^−^ (A) or conventional CD45RA^−^ HELIOS^+^TIGIT^+^ mTregs (B), following IL-2 treatment in T1D patients receiving: (i) the lower IL-2 doses of 0.04–0.045×10^6^ U/ml (N = 2; depicted in black); or (ii) the higher IL-2 doses of 0.6–1.5 x 10^6^ U/ml (N = 6; depicted in red). Data were obtained from intracellular staining of cryopreserved PBMCs from eight selected T1D patients enrolled in the “*Adaptive study of IL-2 dose on regulatory T cells in type 1 diabetes”* (DILT1D). The DILT1D data from individuals prior to normalization as a group are available, however they cannot be anonymized sufficiently to be able to put into the public domain without risk of participant identification. Data are available on request, through the Cambridge University institutional repository (DOI link: https://doi.org/10.17863/CAM.832).

The dataset contained in this article provides a detailed characterization of the expression of the IL-6 receptor (IL-6R) on circulating human CD4^+^ CD127^low^CD25^high^ T cells *ex vivo*. These data also provide a functional characterization of the assessed T cell subsets, with a particular emphasis on a subset of IL-6R^hi^ regulatory T cells (Tregs) lacking the expression of the co-inhibitory receptor TIGIT. The Fig. 1 and Fig. 2 depict the delineation of the assessed immune subsets and their responsiveness to IL-6 signalling. Fig. 3 depicts data from a clinical study investigating the responsiveness of the assessed T cells subsets to IL-2 signalling *in vivo*. Fig. 4 depicts the proliferative capacity of the Treg subsets *in vitro*, in the absence of exogenous IL-2. Fig. 5 and Fig. 6 depict the expression at the protein level of Th17 surface markers. Fig. 7 depicts the differential mRNA expression of 579 immune genes between IL-6R^hi^TIGIT^−^ and IL-6R^hi^TIGIT^+^ Tregs. Fig. 8, Fig. 9 and Fig. 10 depict the immunophenotyping of the Th17 transcription factor RORγt, and different tissue-homing receptors at the protein level. Fig. 11 depicts the expression of two cytokines, IL-17 and IL-10, in TIGIT^+^ Tregs and Fig. 12 depicts the variation of HELIOS^-^TIGIT^-^ and HELIOS^+^TIGIT^+^ Tregs measured by intracellular flow cytometry on cryopreserved peripheral blood mononuclear cells (PBMCs) on 8 selected patients from the DILT1D clinical study. Table 1 provides the complete information on the fluorochrome-conjugated antibody panels used in tis study, and Table 2 and Table 3 contain the complete gene expression data of 579 immune genes on the assessed T cell subsets, obtained in *ex vivo*-isolated cells or following *in vitro* stimulation, respectively.

**Table 1:** Antibodies and immunostaining panels used for flow cytometry. Detailed description of the fluorochrome-conjugated antibodies and immunostaining panels used to immunophenotype and flow sort the assessed CD4^+^ T cell populations.

**Immunostaining Panel**	**Antibody**	**Fluorochrome**	**Clone**	**Manufacturer**
**DILT1D Treg immunophenotyping (Whole blood)**	CD4	AF700	RPA-T4	BioLegend
	CD25^a^	APC	M-A251+2A3	BD Biosciences
	CD127	PE-Cy7	eBioRDR5	eBioscience
	CD45RA	BV785	HI100	BioLegend
	CD14	PB	M5E2	BioLegend
	CCR6	AF488	G034E3	BioLegend
	IL-6R	PE	UV4	BioLegend
	CD8	APC-Cy7	RPA-T8	BioLegend
	CXCR3	PerCP Cy5.5	G025H7	BioLegend
	CD62L	BV605	DREG-56	BioLegend
				
**IL-6R**^**hi**^**Treg immunophenotyping (Freshly isolated PBMCs)**	CD3	BV510	UCHT1	BioLegend
	CD4	BUV398	SK3	BD Biosciences
	CD25^a^	APC	M-A251+2A3	BD Biosciences
	CD127	PE-Cy7	eBioRDR5	eBioscience
	CD45RA	BV785	HI100	BioLegend
	CTLA-4	PE-CF594	BNI3	BD Biosciences
	CD8	APC-Cy7	RPA-T8	BioLegend
	CD62L	BV605	DREG-56	BioLegend
	PD-1	BV711	EH12.2	BD Biosciences
	IL-6R	BV421	M5	BD Biosciences
	HELIOS	FITC	22F6	BioLegend
	Ki-67	PerCP Cy5.5	B59	BD Biosciences
	FOXP3	PE	259D	BioLegend
				
**IL-6R**^**hi**^**TIGIT^-^ Treg immunophenotyping (Freshly isolated PBMCs)**	CD3	BV510	UCHT1	BioLegend
	CD4	BUV737	SK3	BD Biosciences
	CD25^a^	APC	M-A251+2A3	BD Biosciences
	CD127	PE-Cy7	eBioRDR5	eBioscience
	CD45RA	BV785	HI100	BioLegend
	HLA-DR	AF700	L243	BioLegend
	CD8	APC-Cy7	RPA-T8	BioLegend
	CD62L	BV605	DREG-56	BioLegend
	CCR6	BV711	11A9	BD Biosciences
	TIGIT	PerCP eFluor710	MBSA43	eBioscience
	CD161	PE	HP-3G10	BioLegend
	IL-6R	BV421	M5	BD Biosciences
	HELIOS	FITC	22F6	BioLegend
	Ki-67	BUV398	B59	BD Biosciences
	FOXP3	PE-CF594	259D	BioLegend
				
**FACS Sorting (Freshly isolated PBMCs)**	CD3	FITC	UCHT1	BioLegend
	CD4	AF700	RPA-T4	BioLegend
	CD25	APC	M-A251	BD Biosciences
	CD127	PE-Cy7	eBioRDR5	eBioscience
	CD45RA	BV785	HI100	BioLegend
	IL-6R	PE	M5	BD Biosciences
	TIGIT	PerCP eFluor710	MBSA43	eBioscience
	Proliferation dye	eFluor450	–	eBioscience
				
**IL-17/IL-10 production (Freshly isolated CD4**^**+**^**T cells)**	CD3	BV510	UCHT1	BioLegend
	CD4	BUV737	SK3	BD Biosciences
	CD25^a^	APC	M-A251+2A3	BD Biosciences
	CD127	PE-Cy7	eBioRDR5	eBioscience
	CD45RA	BV785	HI100	BioLegend
	TIGIT	PerCP eFluor710	MBSA43	eBioscience
	HELIOS	FITC	22F6	BioLegend
	FOXP3	PE-CF594	259D	BioLegend
	IL-17	AF700	BL168	BioLegend
	IC	AF700	MOPC-21	BioLegend
	IL-10	PE	JES-9D7	BioLegend
	IC	PE	RTK2071	BioLegend
	Viability Dye	eFluor780	–	eBioscience
				
**Chemokine receptor profiling (Freshly isolated PBMCs)**	CD4	BUV737	SK3	BD Biosciences
	CD25^a^	APC	M-A251+2A3	BD Biosciences
	CD127	PE-Cy7	eBioRDR5	eBioscience
	CD45RA	BV785	HI100	BioLegend
	TIGIT	PerCP eFluor710	MBSA43	eBioscience
	CD27	BUV398	L128	BD Biosciences
	CD8	APC-Cy7	RPA-T8	BioLegend
	IL-6R	BV421	M5	BD Biosciences
	CCR4	BV510	L291H4	BioLegend
	CCR6	AF488	G034E3	BioLegend
	CCR10	PE	6588-5	BioLegend
	CXCR3	AF700	G025H7	BioLegend
	CXCR5	PE-CF594	J252D4	BioLegend
	CD15s	BV711	CSLEX1	BD Biosciences
				
**RORγt / gut-homing immunophenotyping (Freshly isolated CD4**^**+**^**T cells)**	CD4	BUV737	SK3	BD Biosciences
	CD25^a^	APC	M-A251+2A3	BD Biosciences
	CD127	PE-Cy7	eBioRDR5	eBioscience
	CD45RA	BV785	HI100	BioLegend
	TIGIT	PerCP eFluor710	MBSA43	eBioscience
	CD3	BV510	UCHT1	BioLegend
	CCR6	BV711	11A9	BD Biosciences
	CD8	APC-Cy7	RPA-T8	BioLegend
	IL-6R	BV421	M5	BD Biosciences
	ITGB7	FITC	FIB504	BioLegend
	HELIOS	FITC	22F6	BioLegend
	ITGA4	BV605	9F10	BioLegend
	CD62L	BV605	DREG-56	BioLegend
	CCR9	PE	L053E8	BioLegend
	RORγ/γt	PE	AFKJS-9	eBioscience
	FOXP3	PE-CF594	259D	BioLegend
	Ki-67	BUV398	B59	BD Biosciences

a Two clones of anti-CD25 that bind to different epitopes were used simultaneously to enhance CD25 staining. IC, Isotype control.

## 2 Experimental design, materials and methods

### 2.1 Patient selection

Patient selection and the protocol for the “Adaptive study of IL-2dose on regulatory T cells in type 1 diabetes” (DILT1D) has been published previously [2], [3]. A subset of 22 T1D patients (median age = 26, range 18–48) were selected for this study, and assessed for the expression of IL-6R on Tregs. A blood sample was taken before treatment to establish baseline Treg frequencies and phenotypes, followed by subcutaneous administration of a single dose of recombinant human IL-2 (Proleukin/aldesleukin; dose range 45,000–737,000 IU/m2) on day 0. The patients were bled 90 min after treatment, and then daily to day 4 and at days 7, 9, 14, 21 and 60.

Study participants for all further immunophenotyping and functional assays included in this study were adult healthy volunteers recruited from the Cambridge BioResource (http://www.cambridgebioresource.org.uk/). All samples were collected after approval from the relevant research ethics committees, and written informed consent was obtained from the participants.

### 2.2 Flow cytometry

Treg immunophenotyping in healthy donors was performed in fresh peripheral blood mononuclear cells (PBMCs) isolated by Ficoll gradient centrifugation (Lymphoprep; STEMCELL Technologies) from whole blood within 2 h of phlebotomy. Cells were stained with fluorochrome-conjugated antibodies against surface receptors (see Table 1 for details) for 45 min at 4 °C. Fixation and permeabilisation was performed using FOXP3 Fix/Perm Buffer Set (BioLegend) and cells were then stained with the respective intracellular antibodies for 45 min at room temperature (Table 1).

For the DILT1D clinical trial participants, 30 ml whole blood were collected into lithium heparin tubes and processed within 4 h of phlebotomy. Immunostaining was performed in whole blood with specific fluorochrome-conjugated antibodies (listed in Table 1) at room temperature for 45 min. IL-6R expression was assessed using a phycoerythrin (PE)-conjugated antibody, which provided the better resolution in our flow cytometric setting. This was critical to increase the sensitivity of the assay, and assess quantitative differences in IL-6R expression in different T cell subsets.

### 2.3 Cell sorting

Cell sorting was performed using a BD FACSAria Fusion flow cytometer (BD Biosciences) after pre-enrichment of CD4^+^ T cells from whole blood by negative selection. Fluorescence-conjugated antibodies used for sorting are described in Table 1. Sorting efficiencies were determined in four donors, based on IL-6R and TIGIT expression and ranged between 90–99%.

### 2.4 Intracellular pSTAT3 immunostainings

PBMCs were isolated from three healthy donors by Ficoll gradient centrifugation from whole blood within 2 h of phlebotomy. IL-6 sensitivity of the memory Treg and Teff subsets was determined by intracellular pSTAT3 immunostaining in freshly isolated PBMCs in response to IL-6 stimulation *in vitro*, as previously described [6].

### 2.5 *in vitro* proliferation assays

To assess the proliferative capacity of IL-6R^hi^TIGIT^−^, IL-6R^hi^TIGIT^+^ and IL-6R^lo^TIGIT^+^ Tregs and memory Teffs, 10^4^ sorted cells from each subset were labelled with eFluor450 Cell Proliferation Dye (eBioscience), and cultured in the presence of exogenous IL-2 (100 U/ml; Proleukin) and anti-CD3/CD28 activation beads, at a 1:1 bead:Teff ratio in X-VIVO 15 + 5% human AB serum. Cells were cultured in X-VIVO 15 + 5% human AB serum for 84 h at 37 °C in V-bottom 96-well cell culture plates (CELLSTAR, Greiner) in the presence of exogenous IL-2 (100 U/ml; Proleukin) and anti-CD3/CD28 activation beads (Life Technologies), at a 1:1 bead:Teff ratio. Proliferation of the responder cells was assessed by the dilution of the proliferation dye by flow cytometry.

Proliferation capacity was calculated using the Division Index (DI) in FlowJo (Tree Star). The DI represents the average number of cell divisions that each seeding Teff cell has undergone and was obtained using the following equation: DI = Total number of Cell Divisions / Initial number of Teff cells in culture = $\left(\left(\frac{G_{1}}{2}\right)*1+\left(\frac{G_{2}}{4}\right)*2+\left(\frac{G_{3}}{8}\right)*3+\left(\frac{G_{4}}{16}\right)*4+\ldots +\left(\frac{G_{n}}{n*2}\right)*n)/(G_{0}+\left(\frac{G_{1}}{2}\right)+\left(\frac{G_{2}}{4}\right)+\left(\frac{G_{3}}{8}\right)+\left(\frac{G_{4}}{16}\right)+\ldots +\left(\frac{G_{n}}{n*2}\right)\right)$, where *n* represents the number of divisions and *G*_(n)_ represents the number of cells that have undergone *n* divisions.

### 2.6 Cytokine secretion assays

To assess cytokine production, CD4^+^ T cells were isolated from whole blood by negative selection using RosetteSep (STEMCELL Technologies) within 2 h of phlebotomy. Cells were resuspended in X-VIVO 15 (Lonza) + 5% heat-inactivated, filtered human AB serum (Sigma), and cultured (1–2×10^6^ CD4s/well) in a 24-well flat-bottom cell culture plate (CELLSTAR, Greiner) at 37 °C in the presence or absence of the 1X Cell Stimulation Cocktail (eBiosiences), containing phorbol myristate acetate (PMA), ionomycin, and protein transport inhibitors (brefeldin A and monensin).

After 6 h culture, cells were harvested and immunostained with surface and intracellular antibodies (listed in Table 1). The unstimulated cells were used to determine background levels of cytokine production. Dead-cell exclusion was performed using the eFluor780 Fixable Viability Dye (eBiosciences).

### 2.7 Transcriptional profiling of the Treg subsets

Gene expression profiling was performed by NanoString, using the pre-designed nCounter Human Immunology v2 Panel (NanoString Technologies). The four assessed immune cell subsets were flow sorted as described above, and 25,000 cells were collected into RLT lysis buffer (Qiagen) either: (i) directly *ex vivo*; or (ii) following *in vitro* stimulation for 165 min in the presence or absence of 50 ng/ml PMA (Sigma-Aldrich) and 500 ng/ml ionomycin (Sigma-Aldrich), without addition of protein transport inhibitors. RNA from the flow-sorted T cell subsets was extracted using the RNAeasy Micro Plus kit (Qiagen), with gDNA cleanup, following manufacturer׳s instructions. Total RNA samples were then hybridized to the NanoString CodeSets, following manufacturer׳s instructions. Expression levels were assessed using an nCounter Flex instrument (NanoString Technologies). Data were processed using the nSolver Analysis Software following normalization of the raw read counts to the geometric mean of positive control spike-ins, and the gene expression of 15 selected housekeeping genes (*ATG10, C14orf166, CD3E, CD46, G6PD, GPI, POLR1B, POLR2A, PSMB5, PSMB10, PTPRC, SDHA, SKI, TOLLIP and TUBB*) that were found to have low variability on both the samples collected *ex vivo* and following *in vitro* stimulation.

### 2.8 Statistical analyses

Statistical analyses were performed using Prism software (GraphPad) and R (www.r-project.org.com). Statistical significance was assessed using a two-tailed non-parametric Mann-Whitney test. Comparison of immune phenotypes between the assessed Treg subsets from the same individual was performed using a two-tailed paired non-parametric Wilcoxon signed rank test.

Differential expression of normalized NanoString transcriptional data was calculated using a paired analysis with DESeq. 2 v1.12.3 [7], with pre-set size factors equal to one for all samples. Adjusted *P* values correspond to the false discovery rates (FDR) for differential expression, computed after correcting *P* values for multiple testing. A missing FDR is reported for genes that were found to contain an expression outlier by DESeq. 2 Cook׳s distance-based flagging of *P* values, and thus excluded from multiple testing.
